# Supplementary material for: Hypothermia improves neuronal network recovery in a human-derived in vitro model of oxygen-deprivation
Source: PLoS One. 2024 Dec 20;19(12):e0314913. doi: 10.1371/journal.pone.0314913 (PMC11661596; doi:10.1371/journal.pone.0314913)
Supplement: S1 Table — Statistical analysis relative to Fig 1. Statistical analysis were performed Two-Way ANOVA with Tukey’s multiple comparisons test. All comparisons with a p-value < 0.05 are shown. (DOCX) [file pone.0314913.s001.docx]

**Supplementary Data**

Elaborate statistical details of figure 1.

| Figure | Panel | Parameter | Comparison | Time point |  | P-value |
| --- | --- | --- | --- | --- | --- | --- |
| *1* | ***e*** | ***MFR*** | Normothermia  vs. hypothermia | 1 h hypoxia | *** | 0.003 |
|  | ***f*** | ***NBR*** | Normothermia  vs. hypothermia | 1 h hypoxia | * | 0.0426 |
|  | ***j*** | ***NBR*** | Normothermia  vs. hypothermia | 6 h recovery | **** | <0.0001 |

Table S1. Statistical analysis relative to Figure 1. Statistical analysis were performed Two-Way ANOVA with Tukey’s multiple comparisons test. All comparisons with a p-value < 0.05 are shown.
