# Supplementary material for: Hypothermia improves neuronal network recovery in a human-derived in vitro model of oxygen-deprivation
Source: PLoS One. 2024 Dec 20;19(12):e0314913. doi: 10.1371/journal.pone.0314913 (PMC11661596; doi:10.1371/journal.pone.0314913)
Supplement: S2 Table — Statistical analysis relative to Fig 2. Statistical analysis were performed Two-Way ANOVA with Tukey’s multiple comparisons test. All comparisons with a p-value < 0.05 are shown. (DOCX) [file pone.0314913.s002.docx]

**Supplementary Data**

Elaborate statistical details of figure 2.

| Figure | Panel | Parameter | Comparison | Time point |  | P-value |
| --- | --- | --- | --- | --- | --- | --- |
| *2* | ***C*** | ***MFR*** | Normothermia  vs. hyperthermia | 2 h hypoxia | * | 0.0196 |
|  |  |  |  | 4 h hypoxia | * | 0.0133 |
|  |  |  |  | 6 h hypoxia | * | 0.0270 |
|  |  |  |  | 8 h hypoxia | * | 0.0393 |
|  |  |  |  | 10 h hypoxia | * | 0.0403 |
|  | ***D*** | ***NBR*** | Normothermia  vs. hyperthermia | 8 h hypoxia | * | 0.0305 |
|  |  |  |  | 10 h hypoxia | ** | 0.0044 |
|  |  |  |  | 12 h hypoxia | *** | 0.0008 |
|  |  |  |  | 14 h hypoxia | **** | <0.0001 |
|  |  |  |  | 16 h hypoxia | **** | <0.0001 |
|  |  |  |  | 18 h hypoxia | **** | <0.0001 |
|  |  |  |  | 20 h hypoxia | **** | <0.0001 |
|  |  |  |  | 22 h hypoxia | **** | <0.0001 |
|  |  |  |  | 24 h hypoxia | **** | <0.0001 |
|  |  |  |  | 26 h hypoxia | *** | 0.0003 |
|  |  |  |  | 28 h hypoxia | ** | 0.0033 |
|  |  |  |  | 30 h hypoxia | ** | 0.0094 |
|  | ***H*** | ***NBR*** | Normothermia  vs. hyperthermia | 6 h recovery | **** | <0.0001 |

Table S2. Statistical analysis relative to Figure 2. Statistical analysis were performed Two-Way ANOVA with Tukey’s multiple comparisons test. All comparisons with a p-value < 0.05 are shown.
