# Supplementary material for: Hypothermia improves neuronal network recovery in a human-derived in vitro model of oxygen-deprivation
Source: PLoS One. 2024 Dec 20;19(12):e0314913. doi: 10.1371/journal.pone.0314913 (PMC11661596; doi:10.1371/journal.pone.0314913)
Supplement: S3 Table — Statistical analysis relative to Fig 3. Statistical analysis were performed Two-Way ANOVA with Tukey’s multiple comparisons test for panels b and c, and Sidak’s multiple comparisons test for panels e and f. All comparisons with a p-value < 0.05 are shown. (DOCX) [file pone.0314913.s003.docx]

**Supplementary Data**

Elaborate statistical details of figure 3.

| Figure | Panel | Parameter | Comparison | Time point |  | P-value |
| --- | --- | --- | --- | --- | --- | --- |
| 3 | B | Dead cells | Normothermia vs. hypothermia | 24h hypoxia | *** | 0.006 |
|  | c | ***Dead cells*** | Normothermia vs. hyperthermia | 48h hypoxia | *** | 0.001 |
|  | f | ***Live cells*** | Normothermia vs. hypothermia | 24h recovery | **** | <0.0001 |
|  |  | ***Apoptotic cells*** | Normothermia vs. hypothermia | 24h recovery | **** | <0.0001 |

Table S3. Statistical analysis relative to Figure 3. Statistical analysis were performed Two-Way ANOVA with Tukey’s multiple comparisons test for panels b and c, and Sidak’s multiple comparisons test for panels e and f. All comparisons with a p-value < 0.05 are shown.
