# Supplementary material for: Hypothermia improves neuronal network recovery in a human-derived in vitro model of oxygen-deprivation
Source: PLoS One. 2024 Dec 20;19(12):e0314913. doi: 10.1371/journal.pone.0314913 (PMC11661596; doi:10.1371/journal.pone.0314913)
Supplement: S4 Table — Statistical analysis relative to Fig 4. Statistical analysis were performed Two-Way ANOVA with Tukey’s multiple comparisons test. All comparisons with a p-value < 0.05 are shown. (DOCX) [file pone.0314913.s004.docx]

**Supplementary Data**

Elaborate statistical details of figure 4.

| Figure | Panel | Parameter | Comparison | Time point |  | P-value |
| --- | --- | --- | --- | --- | --- | --- |
| *4* | ***b*** | ***Synaptic puncta / 10 μm*** | Baseline vs. normothermia | 6 h hypoxia | * | 0.0462 |
|  |  |  | Baseline vs. hypothermia | 6 h hypoxia | **** | <0.0001 |
|  |  |  | Normothermia vs.  Hypothermia | 6 h hypoxia | **** | <0.0001 |
|  |  |  | Hypothermia vs. hyperthermia | 6 h hypoxia | **** | <0.0001 |
|  | ***C*** | ***Synaptic puncta / 10 μm*** | Baseline vs. hypothermia | 24 h hypoxia | *** | 0.0010 |
|  |  |  | Normothermia vs.  hypothermia | 24 h hypoxia | **** | <0.0001 |
|  |  |  | Hypothermia vs.  hyperthermia | 24 h hypoxia | **** | <0.0001 |
|  | ***D*** | ***Synaptic puncta / 10 μm*** | Baseline vs. normothermia | 48 h hypoxia | **** | <0.0001 |
|  |  |  | Baseline vs. hypothermia | 48 h hypoxia | *** | 0.0006 |
|  |  |  | Baseline vs. hyperthermia | 48 h hypoxia | ** | 0.0035 |
|  |  |  | Normothermia vs.  hypothermia | 48 h hypoxia | **** | <0.0001 |
|  |  |  | Hypothermia vs.  hyperthermia | 48 h hypoxia | **** | <0.0001 |
|  | ***F*** | ***Synaptic puncta / 10 μm*** | Normothermia vs. hypothermia | 6h recovery | Ns | >0.9999 |

Table S4. Statistical analysis relative to Figure 4. Statistical analysis were performed Two-Way ANOVA with Tukey’s multiple comparisons test. All comparisons with a p-value < 0.05 are shown.
