# Supplementary material for: Hypothermia improves neuronal network recovery in a human-derived in vitro model of oxygen-deprivation
Source: PLoS One. 2024 Dec 20;19(12):e0314913. doi: 10.1371/journal.pone.0314913 (PMC11661596; doi:10.1371/journal.pone.0314913)
Supplement: S5 Table — Statistical analysis relative to Fig 5. Statistical analysis were performed one-way ANOVA with Dunnet’s multiple comparisons test. All comparisons with a p-value < 0.05 are shown. (DOCX) [file pone.0314913.s005.docx]

**Supplementary Data**

Elaborate statistical details of figure 5.

| Figure | Panel | Parameter | Comparison | Time point |  | P-value |
| --- | --- | --- | --- | --- | --- | --- |
| *5* | ***c*** | ***MFR*** | Baseline vs. Hypothermia | 1 h | **** | <0.0001 |
|  |  |  |  | 2 h | ** | 0.0036 |
|  |  |  |  | 4 h | * | 0.0279 |
|  |  |  |  | 6 h | ** | 0.0029 |
|  |  |  |  | 8 h | *** | 0.0003 |
|  |  |  |  | 10 h | *** | 0.0001 |
|  |  |  |  | 12 h | **** | <0.0001 |
|  |  |  |  | 14 h | **** | <0.0001 |
|  |  |  |  | 16 h | **** | <0.0001 |
|  |  |  |  | 18 h | **** | <0.0001 |
|  |  |  |  | 20 h | **** | <0.0001 |
|  |  |  |  | 22 h | **** | <0.0001 |
|  |  |  |  | 24 h | **** | <0.0001 |
|  |  |  |  | 28 h | **** | <0.0001 |
|  |  |  |  | 30 h | **** | <0.0001 |
|  |  |  |  | 48 h | **** | <0.0001 |
|  | ***d*** | ***NBR*** | Baseline vs. Hypothermia | 1 h | **** | <0.0001 |
|  |  |  |  | 8 h | * | 0.0477 |
|  |  |  |  | 12 h | ** | 0.0023 |
|  |  |  |  | 14 h | *** | 0.0008 |
|  |  |  |  | 16 h | **** | <0.0001 |
|  |  |  |  | 18 h | **** | <0.0001 |
|  |  |  |  | 20 h | **** | <0.0001 |
|  |  |  |  | 22 h | **** | <0.0001 |
|  |  |  |  | 24 h | **** | <0.0001 |
|  |  |  |  | 28 h | **** | <0.0001 |
|  |  |  |  | 30 h | **** | <0.0001 |
|  |  |  |  | 48 h | **** | <0.0001 |
|  | ***e*** | ***NBD*** | Baseline vs. hypothermia | 1 h | * | 0.0276 |
|  |  |  |  | 2 h | *** | 0.0002 |
|  |  |  |  | 4 h | ** | 0.0092 |
|  |  |  |  | 16 h | * | 0.0229 |
|  |  |  |  | 18 h | ** | 0.0015 |
|  |  |  |  | 20 h | **** | <0.0001 |
|  |  |  |  | 22 h | **** | <0.0001 |
|  |  |  |  | 24 h | *** | 0.0001 |
|  |  |  |  | 28 h | *** | 0.0001 |
|  |  |  |  | 30 h | **** | <0.0001 |
|  | ***f*** | ***MFR*** | Baseline vs. Hyperthermia | 22 h | * | 0.0297 |
|  |  |  |  | 24 h | ** | 0.0097 |
|  |  |  |  | 26 h | ** | 0.0043 |
|  |  |  |  | 28 h | *** | 0.0009 |
|  |  |  |  | 30 h | *** | 0.0002 |
|  |  |  |  | 32 h | **** | <0.0001 |
|  |  |  |  | 34 h | **** | <0.0001 |
|  |  |  |  | 36 h | **** | <0.0001 |
|  |  |  |  | 38 h | **** | <0.0001 |
|  |  |  |  | 40 h | **** | <0.0001 |
|  |  |  |  | 42 h | **** | <0.0001 |
|  |  |  |  | 44 h | **** | <0.0001 |
|  |  |  |  | 46 h | **** | <0.0001 |
|  |  |  |  | 48 h | **** | <0.0001 |
|  | ***g*** | ***NBR*** | Baseline vs. hyperthermia | 30 h | * | 0.0141 |
|  |  |  |  | 32 h | ** | 0.0021 |
|  |  |  |  | 34 h | *** | 0.0004 |
|  |  |  |  | 36 h | **** | <0.0001 |
|  |  |  |  | 38 h | **** | <0.0001 |
|  |  |  |  | 40 h | **** | <0.0001 |
|  |  |  |  | 42 h | **** | <0.0001 |
|  |  |  |  | 44 h | **** | <0.0001 |
|  |  |  |  | 46 h | **** | <0.0001 |
|  |  |  |  | 48 h | **** | <0.0001 |
|  | ***h*** | ***NBD*** | Baseline vs. hyperthermia | 18 h | * | 0.0359 |
|  |  |  |  | 20 h | * | 0.0186 |
|  |  |  |  | 22 h | * | 0.0232 |
|  |  |  |  | 24 h | * | 0.00140 |
|  |  |  |  | 26 h | * | 0.0204 |
|  |  |  |  | 28 h | **** | <0.0001 |
|  |  |  |  | 30 h | **** | <0.0001 |
|  |  |  |  | 32 h | **** | <0.0001 |
|  |  |  |  | 34 h | **** | <0.0001 |
|  |  |  |  | 36 h | **** | <0.0001 |
|  |  |  |  | 38 h | **** | <0.0001 |
|  |  |  |  | 40 h | **** | <0.0001 |
|  |  |  |  | 42 h | ** | 0.0029 |
|  |  |  |  | 44 h | * | 0.0432 |
|  |  |  |  | 46 h | ** | 0.0088 |
|  | ***j*** | ***MFR*** | Baseline vs. hypothermia | 6 h recovery | **** | <0.0001 |
|  | ***k*** | ***NBR*** | Baseline vs. hypothermia | 6 h recovery | **** | <0.0001 |
|  | ***l*** | ***MFR*** | Baseline vs. hyperthermia | 6 h recovery | **** | <0.0001 |
|  | ***M*** | ***NBR*** | Baseline vs. hyperthermia | 6 h recovery | **** | <0.0001 |

Table S5. Statistical analysis relative to Figure 5. Statistical analysis were performed one-way ANOVA with Dunnet’s multiple comparisons test. All comparisons with a p-value < 0.05 are shown.
