# Supplementary material for: Hypothermia improves neuronal network recovery in a human-derived in vitro model of oxygen-deprivation
Source: PLoS One. 2024 Dec 20;19(12):e0314913. doi: 10.1371/journal.pone.0314913 (PMC11661596; doi:10.1371/journal.pone.0314913)
Supplement: Attachment — Submitted filename: Response_To_reviewers.docx [file pone.0314913.s006.docx]

**Comments to the Author**

1. Is the manuscript technically sound, and do the data support the conclusions?
The manuscript must describe a technically sound piece of scientific research with data that supports the conclusions. Experiments must have been conducted rigorously, with appropriate controls, replication, and sample sizes. The conclusions must be drawn appropriately based on the data presented.

Reviewer #1: Partly

Reviewer #2: Yes

Reviewer #3: Partly

Reviewer #4: Partly

2. Has the statistical analysis been performed appropriately and rigorously?

Reviewer #1: Yes

Reviewer #2: Yes

Reviewer #3: Yes

Reviewer #4: Yes

3. Have the authors made all data underlying the findings in their manuscript fully available?
The [PLOS Data policy](https://eur02.safelinks.protection.outlook.com/?url=http%3A%2F%2Fwww.plosone.org%2Fstatic%2Fpolicies.action%23sharing&data=05%7C02%7Ce.j.h.f.voogd%40utwente.nl%7C96a2b24601ea4a11d2d208dc92028147%7C723246a1c3f543c5acdc43adb404ac4d%7C0%7C0%7C638545788213706652%7CUnknown%7CTWFpbGZsb3d8eyJWIjoiMC4wLjAwMDAiLCJQIjoiV2luMzIiLCJBTiI6Ik1haWwiLCJXVCI6Mn0%3D%7C0%7C%7C%7C&sdata=C8Una5CtVs7j3g66kUApVcRNt6hhCZK%2Fn1QyhJk%2BhNA%3D&reserved=0) requires authors to make all data underlying the findings described in their manuscript fully available without restriction, with rare exception (please refer to the Data Availability Statement in the manuscript PDF file). The data should be provided as part of the manuscript or its supporting information, or deposited to a public repository. For example, in addition to summary statistics, the data points behind means, medians and variance measures should be available. If there are restrictions on publicly sharing data—e.g. participant privacy or use of data from a third party—those must be specified.

Reviewer #1: Yes

Reviewer #2: Yes

Reviewer #3: Yes

Reviewer #4: No

4. Is the manuscript presented in an intelligible fashion and written in standard English?

PLOS ONE does not copyedit accepted manuscripts, so the language in submitted articles must be clear, correct, and unambiguous. Any typographical or grammatical errors should be corrected at revision, so please note any specific errors here.

Reviewer #1: Yes

Reviewer #2: Yes

Reviewer #3: No

Reviewer #4: Yes

5. Review Comments to the Author

Please use the space provided to explain your answers to the questions above. You may also include additional comments for the author, including concerns about dual publication, research ethics, or publication ethics. (Please upload your review as an attachment if it exceeds 20,000 characters)

**Reviewer #1:**

The manuscript of Voogd et al. presents intriguing data, further confirming the previous finding that hypothermia applied during hypoxia may have neuroprotective effects. The topic is timely and of significant importance, as the prevalence of ischemic stroke is continuously increasing worldwide, however no neuroprotective agents have been demonstrated to impact clinical outcomes.

The authors monitored the electrophysiological activity throughout the hypoxic treatment until the end of the recovery period, thereby providing a complete, comprehensive picture of how temperature affects neuronal activity. However a similar comprehensive tracking of the number of synaptic puncta is missing from the study. In my opinion, it would be highly informative to investigate the number of synaptic puncta at the end of the recovery period as well. Furthermore, there is no information available regarding the viability of cells. Authors mentioned in the discussion, that under hypoxia, hyperthermia may induce apoptosis. Is it possible, that the loss of neuronal network functioning observed in this this study may be due to cell death?

**Synaptic puncta.** We thank the reviewer for his/her comments and we agree that the extension of the time points of synaptic puncta is highly informative. Therefore we added data on synaptic puncta at 6 and 24 hours after recovery for normothermic and hypothermic neuronal networks. We found at 24 hours of recovery a significantly higher number of synaptic puncta in hypothermic neuronal networks as compared to normothermic. We added these additional results to Figure 4 (e-g) and the following text:

Line 360-366: “*Next, we examined the recovery of neuronal networks under normothermic and hypothermic conditions, excluding hyperthermic networks because of the absence of functional recovery after hypoxia (fig. 4 e-g). After 6 hours of recovery, normothermic and hypothermic neuronal networks had 0.84 ± 0.07 synaptic puncta per 10 μm and 0.99 ± 0.08 synaptic puncta per 10 μm , respectively (p > 0.9999). After 24 hours of recovery, hypothermic neuronal networks had a higher number of synaptic puncta per 10 μm than normothermic neuronal networks (1.6 ± 0.11 vs. 0.8 ± 0.09; p<0.0001).*

**Fig. 4.** **Number of** **synaptic puncta increased during and after hypoxia in hypothermic neuronal networks**. **e.** Representative images showing synaptic puncta (arrowheads, positive for synapsin 1/2; red) along the dendrite (positive for MAP2; green; nuclei positive for DAPI; blue) in normothermic and hypothermic neuronal networks after 6 and 24 hours of recovery. Scale bar: 10 μm. **f-g.** Bar graphs showing the number of synaptic puncta per 10 μm dendrite in neurons exposed to normothermia (n=4) or hypothermia (n=6) after **f.** 6 hours and **g.** 24 hours of recovery. Black line represents baseline number of synaptic puncta (n = 11). *P < 0.05, **P < 0.005, ***P < 0.0005, ****P < 0.0001. Two-way ANOVA with Tukey’s multiple comparison analyses were performed between conditions. Exact p values are reported in table S3. “

**Live, dead and apoptotic cells.** We agree with the reviewer that the viability of the neuronal networks during the different temperature regimens and hypoxia holds valuable information. Therefore, we added data on cell viability including apoptosis after 24 and 48 hours of hypoxia for normothermic, hypothermic and hyperthermic neuronal networks, as well as 6 and 24 hours of recovery for normothermic and hypothermic neuronal networks. We found that, after 24 hours of hypoxia, neuronal networks treated with hypothermia had significantly fewer dead cells compared to normothermia. Hyperthermic networks showed similar cell viability as normothermia. After 48 hours of hypoxia, neuronal networks treated with hypothermia showed no significant differences in cell viability compared to normothermic networks. Hyperthermic networks showed significantly less live cells and more dead cells compared to normothermic networks. During recovery, we found after 6 hours, normothermic networks had significantly more live cells compared to hypothermic networks. However, after 24 hours of recovery we found that hypothermic networks had significantly better outcomes, by showing more live cells, and less apoptotic cells.

The results are reported in a new figure (new Figure 3). We added the following paragraph to the results, and the complete description of the methodology for the identification of live, dead and apoptotic cells in the Material and Methods section (line157-180).

Line 310-344: *“****Hypothermia improves cell viability after hypoxia***

*We next investigated the effect of normo-, hypo-, and hyperthermia on cell viability (Fig. 3a-b). After 24 hours of hypoxia, under normothermia we observed 36% live cells, 11% apoptotic cells, and 52% dead cells. Under hypothermia, there was a slightly higher percentage of live cells (48%) and apoptotic cells (19%), with significantly fewer dead cells (34%) compared to normothermia (p = 0.0006). Hyperthermia resulted in 34% live cells, 10% apoptotic cells, and 56% dead cells, showing a similar pattern to normothermia with no significant differences in live, apoptotic or dead cell counts.*

*After 48 hours of hypoxia, hypothermic networks maintained a higher percentage of live cells (61%) compared to normothermic networks (55%), and fewer dead cells (22% vs. 32%), although this difference was not statistically significant. Hyperthermia was associated with a significant decline in cell viability, exhibiting the lowest percentage of live cells (33%) and the highest percentage of dead cells (63%). Significant differences were found between normothermic and hyperthermic networks in both live cell count (p = 0.0129) and dead cell count (p = 0.0001).*

*Next, we examined the recovery of neuronal networks under normothermic and hypothermic conditions, excluding hyperthermic networks because of the absence of functional recovery after hypoxia (fig. 3d-f). After 6 hours of recovery, under normothermia, neuronal networks showed a higher percentage of live cells (62%) compared to hypothermic networks, although not statistically significant. However, after 24 hours of recovery, hypothermic neuronal networks demonstrated significantly better outcomes, with 93% live cells compared to 37% in normothermic networks (p < 0.0001). Additionally, hypothermic networks had significantly fewer apoptotic cells (16%) compared to normothermic networks (49%; p < 0.0001).”*

*Fig. 3* ***Hypothermia improves cell viability after hypoxia. a.*** *Representative images showing cell viability including apoptosis of neuronal networks exposed to 24 or 48 hours hypoxia and normothermia, hypothermia or hyperthermia. Neuronal networks stained for DAPI (blue), Cell event Caspase-3/7 (green) and propidium iodine (red). Scale bar 30 μm.* ***b-c.*** *Stacked bar graphs showing the percentage of live, apoptotic (apo) and dead cells after* ***b.*** *24 hours of hypoxia and* ***c.*** *48 hours of hypoxia for normothermia (n=9), hypothermia (n=7) and hyperthermia (n=3).* ***d.*** *Representative images showing cell viability including apoptosis of neuronal networks exposed to 6 or 24 hours of recovery after hypoxia and normothermia or hypothermia. Neuronal networks were stained for DAPI (blue), Cell event Caspase-3/7 (green) and propidium iodine (red).* ***e-f.*** *Stacked bar graphs showing the percentage of live, apoptotic (apo) and dead cells after* ***e.*** *6 hours of recovery or* ***f.*** *24 hours of recovery for normothermia (n=4) and hypothermia (n=6). *P < 0.05, **P < 0.005, ***P < 0.0005, ****P < 0.0001. Two-way ANOVA with* ***b-c.*** *Tukey’s multiple comparisons and e-f. Sidak’s multiple comparisons test. “*

**Reviewer #2:**

The authors established a neuronal network model from hiPSC for MEA neuronal activity test in their previous publication. In this study, they used this model to investigate the effects of hypothermia and hyperthermia on neuronal activity after hypoxia.

1. In the title, “in vitro model of the ischemic penumbra” is incorrect. In the penumbra, blood flow is typically reduced to 30%-70%. The authors used 2% hypoxia which provides too little oxygen but too much glucose to accurately mimic penumbra situation in vitro. A modified oxygen-glucose deprivation (OGD) model with 5-8% hypoxia and 30% glucose content is recommended.

We thank the reviewer for his / her comment. We realize that the term "penumbra" was not directly addressed until the end of the discussion. Our model mimics oxygen deprivation in general, which is a key feature of the ischemic penumbra. To better reflect the scope of our work, we adjusted the title to: “*Hypothermia improves neuronal network recovery in a human-derived in vitro model of oxygen-deprivation*”.

We are aware that the level of oxygen used in this study (2% O_2_) is not in line with what is observed in vivo. This type of oxygen-deprivation is essential for replicating the conditions under which neuronal networks become electrically silent over time, yet remaining potentially salvageable upon restoration of normoxia, which is a key characteristic of the ischemic penumbra. With higher oxygen levels, the neuronal networks remain functionally active (as also shown in a rodent in vitro model [1]), which hampers testing of neuroprotective investigations. While glucose was not lowered in our initial setup, our previous studies have shown that long-term hypoxia results in decreased glucose levels by consumption [2, 3], aligning it with in vivo penumbral conditions, where glucose is also reduced by consumption. Thus, we believe that our results can be relevant for ischemic stroke research.

2. MEA technology has been utilized for over two decades, allowing numerous researchers to study neuronal activity under various physiological and pathological conditions, such as hypothermia, hyperthermia, and hypoxic stress, using either rodent, sheep or human neurons (e.g., PMID: 17081617; PMID: 17093117; PMID: 34975426; PMID: 32320794). It would be informative if the authors used patient-derived neurons, despite the difficulty in obtaining them from ischemic patients. Or, alternatively they may at least use primary human neurons from other diseases. iPSC-derived neurons remain quite artificial, and their results do not provide significant new insights in this field. It is already known that hypothermia is neuroprotective and promotes the recovery of neuronal activity after hypoxia, but it has the opposite effects under normal conditions. Conversely, hyperthermia is known to exacerbate neuronal damage after hypoxia.

We acknowledge that the use of microelectrode array (MEA) technology has indeed been extensive and highly informative in studying neuronal activity under various conditions. However, the suggestion to use patient-derived neurons or primary human neurons from other diseases is, while valuable, beyond the scope of our current study for several reasons. Using patient-derived neurons, especially from stroke patients, would require a significant shift in resources, methodology, and scope, as well as approval of ethical committees. It would be a whole new study in terms of research objectives and experimental design, which is beyond the boundaries of our current study's aims. We agree that patient-derived neurons and primary human neurons are valuable models for translational research. However, we believe that using well-characterized healthy induced pluripotent stem cell (iPSC)-derived neurons provides a valuable first step, by providing a controlled and reproducible system to investigate the effects of hypoxia and temperature regimens [4, 5]. These cells allow us to focus on the electrophysiological responses to hypoxia and hypothermia/hyperthermia without the additional complexities introduced by disease-specific or patient-specific variations, thereby allowing us to contribute meaningful and reproducible data to the field.

3. To enhance the novelty of the present study, I recommend that the authors test the effects of different temperatures and oxygen levels in their model. The author noted that in some reports, even 36°C is neuroprotective after hypoxia. Providing a systematic view of temperature effects could be valuable for therapeutic applications.

We appreciate the reviewer's suggestion to test the effects of different temperatures and oxygen levels in our model. The effect of different oxygen levels has already been studied in detail [1]. Based on those findings, we selected the oxygen level that strikes a balance between inducing a significant loss of neuronal functionality and providing a window for evaluating neuroprotective strategies. This level allows us to model conditions where neuronal networks are impaired but not irreversibly damaged, ensuring that interventions such as hypothermia can still offer protective effects, rather than inducing widespread cell death.

It would be interesting to investigate a broader range of temperatures, but this would require a significant number of additional experiments. That goes beyond the current scope of our study. Our primary goal was to determine whether hypothermia could offer neuroprotection and whether hyperthermia would exacerbate neuronal damage in our in vitro model.

The temperatures we selected —34°C for hypothermia and 39°C for hyperthermia— were based on values reported in the literature that are clinically relevant. We already observe significant protective effects at 34°C, and further temperature testing, although valuable in its own right, would not necessarily provide novel insights for the specific aims of our study. Furthermore, while we see a clear neuroprotective effect at hypothermia at 34°C, these findings are not intended to prescribe a precise temperature range for clinical application, since such a translation from in vitro to in vivo is challenging. The main takeaway for therapeutic purposes is that hypothermia is neuroprotective by synapse recovery and improved cell viability and not that a specific temperature should be used.

Given these considerations, we believe the temperatures we chose effectively demonstrate the protective role of hypothermia and the deleterious role of hyperthermia. Further testing is outside the intended focus of this research.

**Reviewer #3:**

About the manuscript PONE-D-24-12582, entitled ”Hypothermia improves neuronal network recovery in a human-derived in vitro model of the ischemic penumbra”, by Eva JHF et al., submitted to PLOS ONE.

While I consider it interesting, the manuscript is rather difficult to read. I would suggest to improve the whole presentation, clarifying the presentation of the results and very much the discussion.

We thank the reviewer for considering our work interesting. We used the reviewer’s suggestions to improve the readability of the manuscript. See responses below for a more detailed explanation of these improvements.

General
The manuscript reports experiments with neuronal networks derived from human induced pluripotent stem cells, monitored with micro-electrode arrays, to investigate how treatment with normothermia, hypothermia and hyperthermia during hypoxia influences neuronal functionality, improving post-hypoxic recovery. The neuronal networks are also evaluated with immunocytochemistry, studying the effect of the treatment on synaptic puncta.

Methods
The description of the methods is detailed, but not well organised, including experimental steps that are not considered when describing the results. The authors describe the protocol for identifying excitatory and inhibitory neurons, but the phenotype is not confirmed nor used for evaluating the effects of hypoxia and/or temperature conditions.

We re-organized the method section to make it clearer and more in line with the presentation of the results. Textual changes are in red in the manuscript (lines 69-222).

We apologize for the confusion in our Material and Methods section. We have not described the methodology for identifying excitatory and inhibitory neurons, but the protocol to differentiate such cell types from hiPSCs. In this study, we indeed included both excitatory and inhibitory neurons because their combined activity significantly influences overall neuronal functionality, providing a more physiologically relevant representation of neuronal populations in the brain, though not identical to in vivo conditions.

Our primary focus in this work was on general network activity and functional readouts, such as electrophysiological responses and synaptic puncta analysis, rather than dissecting the specific contributions of excitatory and inhibitory neurons. However, differential effects of hypoxia on these cells has been shown, with inhibitory neurons showing higher vulnerability [6]. Thus, we acknowledge the interest in exploring differences between these neuronal subtypes, and future research could further investigate how excitatory and inhibitory neurons specifically respond to hypoxia and temperature variations.

Results
While the experimental timing is schematically described in Fig. 1, 2 and 4, it is not clear enough when the recording is performed. Is it continuously performed, as indicated in Fig.1e-g? But, when are the samples taken for building the graphics at Fig. 1i-k? Please, clarify that for all figures, completing and making it uniform along the figure legends.

We apologize for being unclear in the depiction of the recording timing. Recordings for graphs containing temporal evolutions were performed for 10 minutes every 2 hours. The recordings for the recovery phase were performed for 10 minutes after 6 hours of re-establishment of normoxia and normothermia. We now made the figures uniform.

To improve clarity, we adapted the figure captions adding information regarding the timing of recordings (i.e., *recorded for 10 minutes every 2 hours / after 6 and 24 hours of recovery*).

Would one understand that the sampling in Fig. 1d, Fig.2b and Fig.4b was taken at the beginning or at the end of a period of 6h, 24h or 48h of asphyxia? Under continuous either normothermia, hypothermia or hyperthermia?, but a final 6h normoxia/normothermia period was also performed, at least for Fig. 4? Please, uniform the layout of the figures. In Fig. 1i-k; Fig. 2g-I and Fig. 4i-l, why not using the same scales? The dotted line represents 100% or 1? Also, in Fig. 4k?

All samples were taken at the end of each respective period, meaning after 6h, 24h, and 48h of hypoxia under continuous normothermia, hypothermia, or hyperthermia conditions. Additionally, we now include data on the recovery of synaptic puncta following 6h or 24h of recovery (normoxia/normothermia). For the recovery phase, recordings were done 6 hours after re-establishing physiological conditions (i.e., normothermia and normoxia), and for immunocytochemistry fixation was done after 6 hours recovery followed by another set of recordings and fixation 18 hours later (adding up to a total of 24h recovery). We adapted the figure legend for more clarity.

We have standardized the layout of all figures, ensuring consistent scales across Fig. 1i-k, Fig. 2g-i, and Fig. 4i-l. In addition, we clarified that the dotted line represents 1 (baseline) in all relevant panels, including Fig. 4k.

We normalized the data, with the maximum value set to 1. Therefore, when we state 50% of baseline, it corresponds to 0.5 of the normalized value. In this context, 0.5 of 1 represents a 50% loss of e.g. mean firing rate.

The timing for Fig. 3 has to be clarified, making possible for the reader to rapidly understand when the tissue was fixed. After 6h, 24h and 48h of hypoxia? The labelling of synapsin ½ is not evident, at least in the figure I received. The scale bar in Fig. 3a is hardly seeing.

We apologize that figure 3 was not clear. We added to the figure caption:

Line 370-373: “*b-d. Bar graphs showing the number of synaptic puncta per 10 μm dendrite in neurons exposed to hypoxia combined with normothermia (black; n=6), hypothermia (blue; n=2) or hyperthermia (red; n=4)* ***after*** *b. 6 hours, c. 24 hours and d. 48 hours. Black line represents baseline number of synaptic puncta (n = 11)”*

We removed the control bar and depicted these values as a horizontal line to improve readability. Furthermore, we improved the quality of figure 3a.

Before evaluating the temperature variable, please, describe clearly on the effect of hypoxia, I guess Control versus Normothermia, describing the time course. Clarify, please, the issue of the effect of 48h of hypoxia, inducing only 50% of decrease of synaptic puncta?
Is that a severe or mild effect? How should be that when comparing with in vivo models? Any specific effect on excitatory or inhibitory neurons, or neurons versus glial cells?

We added to the Discussion a section on the effect of hypoxia on neuronal networks in normothermia.

Line 429-441: “***Hypoxia is deleterious for network functioning and cell viability in neuronal networks****. Our results showed that hypoxia led to a persistent decrease in neuronal functionality, synaptic puncta and cell viability. Furthermore, subsequent re-oxygenation led to partial recovery in these readouts. This was in line with earlier studies [37, 44]. After the hypoxic period, we found that the number of synaptic puncta was reduced by half. Synaptic failure leading to a reduction of functional synapses, and ultimately loss of synapses, is a known phenomenon of hypoxic injury in neurons [42, 45-49]. This effect of hypoxia on neurons leads to compromised neuronal function [50], eventually leading to decreased viability [37]. The remaining synapses may still contribute to plasticity, offering potential for recovery once the hypoxic stress subsides. Additionally, while our study focused on overall synaptic puncta, distinguishing between excitatory and inhibitory synapses could provide further insight into cell-type-specific vulnerabilities. A prior study has shown that inhibitory neurons are more vulnerable to hypoxic stress, leading to an increased excitation/inhibition ratio [51].”*

A 50% decrease in synaptic puncta represents a significant loss of neuronal connectivity. It should be noted that our study focused on synaptic puncta in general, without distinguishing between excitatory or inhibitory synapses or the specific impact on neurons versus glial cells, which could provide further insights into the selective vulnerability or resilience of different cell types. It has been previously found that inhibitory neurons are more vulnerable to hypoxic stress leading to an increased excitation / inhibition ratio [6].

Discussion
I would recommend to start by discussing the relevance and advantages of the experimental mode, also summarising the obtained results, making clear what are we learning with the results. Also, please, explain about the relevance of the effects observed after hypothermia, producing outcomes surpassing the control condition? How long does it take for seeing a deleterious effect of hyperthermia? 48h (as seeing in Fig.3b-d??

We added the relevance and advantages of the experimental model, as well as a summary of the results, in the discussion.

Line 413-427: “*In this study, we used a validated human in vitro model to investigate the vulnerability of neuronal networks to hypoxia [2].* *In addition to the human nature, advantages of the model include precise control over environmental conditions, such as oxygen levels and temperatures, providing a clear understanding of how these variables influence neuronal network functions, synaptic integrity, cell viability and recovery potential.*

*Our findings show that hypothermia (34°C) during hypoxia stabilized NBR and slowed their decline. The 1.7-fold increase in NBR compared to baseline suggests enhanced recovery of neuronal networks under hypothermic conditions. This result was further supported by an increased number of synaptic puncta in neuronal networks subjected to hypothermia during and after hypoxia, as well was improved cell viability after hypoxia. In contrast, hyperthermia (39°C) initially elevated NBR, followed by a rapid decline. After 6 hours of reoxygenation, networks exposed to hyperthermia exhibited pronounced impairments, with minimal MFR and absent NBR. These networks also showed a persistent loss of synaptic puncta and cell viability. These results suggest that hypothermia may protect neuronal networks during hypoxia and improve recovery, whereas hyperthermia exacerbates functional impairments.”*

Based on the data presented in Fig. 3b-d, the deleterious effects of hyperthermia on neuronal networks begin to manifest within the first 6 hours of hypoxia. As shown in Fig. 3b, synaptic puncta are already significantly reduced under hyperthermic conditions compared to normothermic and hypothermic conditions. This suggests that hyperthermia exerts an early damaging effect on synaptic connectivity. By 24 hours (Fig. 3c), this reduction in synaptic puncta persists, and by 48 hours (Fig. 3d), the deleterious impact of hyperthermia is most pronounced, with a marked loss of synaptic puncta. Therefore, while the damage starts as early as 6 hours, the full extent of hyperthermia-induced impairments becomes increasingly evident over the 48-hour hypoxic period.

Discuss please about the physiological relevance of the obtained results. What is the relevance of an increase in synaptic puncta following 48h hypoxia + hypothermia?

The relevance of an increase in synaptic puncta following 48 hours of hypoxia under hypothermia lies in the potential restorative mechanisms of synaptic homeostasis. Synaptic puncta represent the points of communication between neurons, and maintaining or increasing their number is crucial for ensuring neuronal connectivity, especially under stress conditions like hypoxia.

This increase suggests that hypothermia may facilitate the preservation or restoration of synaptic connections, supporting neuronal network functionality. This is particularly important because, during hypoxia, neurons are highly vulnerable to losing their connections, which can lead to a cascade from functional impairments to cell death. By increasing synaptic puncta, hypothermia may help maintaining the integrity of neuronal communication pathways, allowing for better functional recovery post-hypoxia and offering a therapeutic benefit in preventing further injury or loss of function in neurons.

We added to the discussion:

*Line 450-453:“The increase in synaptic puncta suggests that hypothermia may promote synaptic homeostasis, preserving or restoring synaptic connections critical for maintaining neuronal communication during metabolic stress. This compensatory mechanism likely supports neuronal survival and functional recovery [56].”*

In general, the discussion is floppy, referring to issues that are not considered by the selected experimental protocol. I would suggest to concentrate on the observed results, expanding then on their relevance, physiologically and/or clinically.
The discussion in lines 382-396 should be improved, expanding the idea that hypothermia would reinforce inhibitory synapses? How is that? considering what is shown in Fig. 3 and 4? What’s about effects on excitatory and/or inhibitory neurons?

We have adapted the discussion following the reviewer’s comment, removing all issues not related to our protocol and results. Changes are highlighted in red in the manuscript.

We did not specifically investigate the effects of hypothermia on excitatory and inhibitory neurons in our model. However, it has been shown that inhibitory neurons are generally more vulnerable to hypoxic conditions, suggesting different susceptibilities between excitatory and inhibitory neurons under metabolic stress [6].

In normoxia, previous studies have demonstrated that hypothermia enhances synaptic stability by increasing levels of the inhibitory postsynaptic scaffolding protein Gephyrin, thereby strengthening GABAA receptor recruitment to inhibitory synapses. This effect is thought to occur rapidly, driven by actin cytoskeleton reorganization rather than protein biosynthesis. Hypothermia’s role in promoting structural plasticity, particularly by enhancing inhibitory signaling, may explain the improved neuronal network recovery we observed following hypothermia [7].

At this stage, we do not have data on how hypothermia in hypoxia differentially affects excitatory and inhibitory neurons or synapses, and thus we refrain from speculating on these dynamics. Additionally, Figures 3 and 4 are not directly comparable, as they reflect different experimental conditions. While hypothermia has been shown to reinforce inhibitory synapses in normoxic environments, inhibitory neurons are more vulnerable under hypoxic conditions. However, we have not examined the differential impact of temperature on excitatory vs. inhibitory synapses in our study, and this remains an area for future investigation.

How to deal with the discussion expressed in line 406-412 and the effects shown in Fig. 2?

We believe this is a misunderstanding and apologize for being unclear. The relevant part of the discussion is about the condition hyperthermia in normoxia, and not hyperthermia in hypoxia, as depicted in figure 2.

We added extra headings to the discussion to make the difference between the sections on temperature + hypoxia and temperature in normoxia clearer.

Line 495: “*Hyperthermia in normoxia is deleterious for neuronal network activity”*

I would suggest that the authors focus on what was observed in the study.
The issue of a therapeutic strategy before a clinical outcome could perhaps deserve some commentaries.

We agree with the reviewer and reduced speculation on issues that we did not investigate.

We removed the following parts from the discussion:

*“Other neurophysiological effects of hypothermia have been studied, as well. In an in vitro model of oxygen and glucose deprivation (OGD), post-OGD hypothermia (33 °C) decreased cell death, reduced intracellular calcium overload, inhibited reactive oxygen species formation, and stabilized mitochondrial membrane potential. Notably, this study showed that the neuroprotective effects of hypothermia in the recovery phase were duration-dependen[60]. Furthermore, hypothermia has also been shown to reduce nitric oxide and superoxide formation, which was likely through NMDA receptor activation [61]. Previous studies have also indicated that the conservation of ATP could play a crucial role in the neuroprotective effects of hypothermia on hypoxic neurons[56, 62-64]. Conservation of ATP by reducing metabolic demand is a potential explanation for our observations.”*

*“Furthermore, hypothermia exerts a modulatory influence on neuronal function by affecting the behavior of voltage-gated membrane channels (i.e., potassium and sodium), leading to a decrease in neuronal firing [68-70].”*

The conclusion should be more convincing. Hypothermia should prevent synaptic loss or rather increase functional efficacy?

We rephrased the conclusion that now also contains the conclusion of the new results of recovery in synaptic puncta and cell viability.

Line 524-531: “*Our study demonstrates that hypothermia, applied during hypoxia, significantly enhances post-hypoxic functional recovery in neuronal networks, likely through the preservation of synaptic integrity and improved cell viability. The improvement in neuronal function, synaptic puncta and survival underscores hypothermia's potential as an effective neuroprotective strategy. These findings suggest that hypothermia could play a critical role in preserving brain function during the vulnerable period between arterial occlusion and recanalization in ischemic stroke patients, offering a promising therapeutic approach to mitigate irreversible neuronal damage.”*

The final sentence “freezing the penumbra” is justifying the title? Indeed, the issue of penumbra is just taken at the end of the article. How should the chosen experimental approach be a model for describing what is occurring in the penumbra? Is the issue of “penumbra” justified for being inserted in the title?

We agree that the term "penumbra" is mainly discussed at the end of the manuscript. Our model mimics oxygen deprivation, which is a key feature of the ischemic penumbra. To better reflect the scope of our work, we have revised the title to "*Hypothermia improves neuronal network recovery in a human-derived in vitro model of oxygen deprivation*”.

In addition, we adapted the conclusion as reported in the previous point.

**Reviewer #4:**

Review of “Hypothermia improves neural network recovery…”

This manuscript evaluates the effects of hypothermic (34C) and hyperthermic (39C) conditions on human-derived neuronal network plated on microelectrode arrays during the application of hypoxia. Functional readouts are performed prior, during and after application of 48hrs of hypoxic conditions at normothermic, hypothermic and hyperthermic temperatures. The results show that the application of hypothermia had some beneficial effects on the network’s electrophysiology, specifically the firing and burst rate whereas the application of hyperthermia had a generally significantly worse outcome than normothermia.

The manuscript is generally well-written and organized, and the data presented is by en large clear. The study is somewhat limited in its focus (primarily focusing on electrical activity and 2 antibody stains), and I have a few comments I’d like the authors to address before I recommend this manuscript to be published:

1. While the data in Fig. 1 shows that there is a clear benefit of applying hypothermic (34C) conditions to the neuronal networks during under hypoxic conditions - it is unclear how viable the culture is 24hrs or even 48hrs after the application of hypothermia. It would significantly strengthen the paper if the authors could provide data further out then just 6hrs (see for example Scimone et al. PlosOne, 2020 - application of hypothermia to TBI).

We agree with the reviewer that the viability of the neuronal networks during the different temperature regimens and hypoxia holds valuable information. Therefore we added data on cell viability including apoptosis after 24 and 48 hours of hypoxia for normothermic, hypothermic and hyperthermic neuronal networks, as well as 6 and 24 hours of recovery for normothermic and hypothermic neuronal networks. We found that after 24 hours of hypoxia, neuronal networks treated with hypothermia had significantly fewer dead cells compared to normothermia. Hyperthermic networks showed similar cell viability as normothermia. After 48 hours of hypoxia, neuronal networks treated with hypothermia showed no significant differences in cell viability compared to normothermic networks. Hyperthermic networks showed significantly less live cells and more dead cells compared to normothermic networks. During recovery, we found that after 6 hours normothermic networks have significantly more live cells compared to hypothermic networks. However, after 24 hours of recovery we found that hypothermic networks have significantly better outcomes, by showing more live cells, and less apoptotic cells.

The results are reported in a new figure (new Figure 3). We added the following paragraph in the results, and the complete description of the methodology for the identification of live, dead and apoptotic cells in the Material and Methods section (line163-178).

Line 310-344: *“****Hypothermia improves cell viability after hypoxia***

*We next investigated the effect of normo-, hypo-, and hyperthermia on cell viability (Fig. 3a-b). After 24 hours of hypoxia, under normothermia, we observed 36% live cells, 11% apoptotic cells, and 52% dead cells. Under hypothermia, there was a slightly higher percentage of live cells (48%) and apoptotic cells (19%), with significantly fewer dead cells (34%) compared to normothermia (p = 0.0006). Hyperthermia resulted in 34% live cells, 10% apoptotic cells, and 56% dead cells, showing a similar pattern to normothermia with no significant differences in live, apoptotic or dead cell counts.*

*After 48 hours of hypoxia, hypothermic networks maintained a higher percentage of live cells (61%) compared to normothermic networks (55%), and fewer dead cells (22% vs. 32%), although this difference was not statistically significant. Hyperthermia was associated with a significant decline in cell viability, exhibiting the lowest percentage of live cells (33%) and the highest percentage of dead cells (63%). Significant differences were found between normothermic and hyperthermic networks in both live cell count (p = 0.0129) and dead cell count (p = 0.0001).*

*Next, we examined the recovery of neuronal networks under normothermic and hypothermic conditions, excluding hyperthermic networks due to the absence of functional recovery after hypoxia (fig. 3d-f). After 6 hours of recovery, under normothermia neuronal networks showed a higher percentage of live cells (62%) compared to hypothermic networks, although not statistically significant. However, after 24 hours of recovery, hypothermic neuronal networks demonstrated significantly better outcomes, with 93% live cells compared to 37% in normothermic networks (p < 0.0001). Additionally, hypothermic networks had significantly fewer apoptotic cells (16%) compared to normothermic networks (49%; p < 0.0001).”*

*Fig. 3* ***Hypothermia improves cell viability after hypoxia. a.*** *Representative images showing cell viability including apoptosis of neuronal networks exposed to 24 or 48 hours hypoxia and normothermia, hypothermia or hyperthermia. Neuronal networks stained for DAPI (blue), Cell event Caspase-3/7 (green) and propidium iodine (red). Scale bar 30 μm.* ***b-c.*** *Stacked bar graphs showing the percentage of live, apoptotic (apo) and dead cells after* ***b.*** *24 hours of hypoxia and* ***c.*** *48 hours of hypoxia for normothermia (n=9), hypothermia (n=7) and hyperthermia (n=3).* ***d.*** *Representative images showing cell viability including apoptosis of neuronal networks exposed to 6 or 24 hours of recovery after hypoxia and normothermia or hypothermia. Neuronal networks were stained for DAPI (blue), Cell event Caspase-3/7 (green) and propidium iodine (red).* ***e-f.*** *Stacked bar graphs showing the percentage of live, apoptotic (apo) and dead cells after* ***e.*** *6 hours of recovery or* ***f.*** *24 hours of recovery for normothermia (n=4) and hypothermia (n=6). *P < 0.05, **P < 0.005, ***P < 0.0005, ****P < 0.0001. Two-way ANOVA with* ***b-c.*** *Tukey’s multiple comparisons and e-f. Sidak’s multiple comparisons test. “*

2. The statement on Line 427 that hypothermia enhances functional recovery during hypoxia might be misleading if the long-term outcome of the culture is unaltered, i.e., if the long-term (24-48hr) outcome does not show any statistical significant effect from normothermia under hypoxic conditions.

We added results on cell viability (see answer to previous point) and synaptic puncta 6 and 24 hours post-hypoxia. We found that, at 24 hours of recovery, there is a significantly higher number of synaptic puncta in hypothermic neuronal networks as compared to normothermic. We added the new results in Figure 4 (e-g) and the following text:

Line 360-366: “*Next, we examined the recovery of neuronal networks under normothermic and hypothermic conditions, excluding hyperthermic networks because of the absence of functional recovery after hypoxia (fig. 4 e-g). After 6 hours of recovery, normothermic and hypothermic neuronal networks had 0.84 ± 0.07 synaptic puncta per 10 μm and 0.99 ± 0.08 synaptic puncta per 10 μm , respectively (p > 0.9999). After 24 hours of recovery, hypothermic neuronal networks had a higher number of synaptic puncta per 10 μm than normothermic neuronal networks (1.6 ± 0.11 vs. 0.8 ± 0.09; p<0.0001).*

**Fig. 4.** **Number of** **synaptic puncta increased during and after hypoxia in hypothermic neuronal networks**. **e.** Representative images showing synaptic puncta (arrowheads, positive for synapsin 1/2; red) along the dendrite (positive for MAP2; green; nuclei positive for DAPI; blue) in normothermic and hypothermic neuronal networks after 6 and 24 hours of recovery. Scale bar: 10 μm. **f-g.** Bar graphs showing the number of synaptic puncta per 10 μm dendrite in neurons exposed to normothermia (n=4) or hypothermia (n=6) after **f.** 6 hours and **g.** 24 hours of recovery. Black line represents baseline number of synaptic puncta (n = 11). *P < 0.05, **P < 0.005, ***P < 0.0005, ****P < 0.0001. Two-way ANOVA with Tukey’s multiple comparison analyses were performed between conditions. Exact p values are reported in table S3. “

3. It would be very helpful for all figures showing time on the x-axis (e.g., Fig.1 e-g, etc) to show the full time data (10min initially, 48 hrs + 6 hrs post) and delineate the different regimes (with shaded regions for example) clearly on the plots. Please also add raster plots for the 6hrs post hypoxia for all groups.

We have chosen to present the hypoxia and recovery periods in separate graphs to maintain clarity. During hypoxia, we focused on the temporal evolution by recording 10-minute intervals every 2 hours over a 48-hour period. In contrast, for the recovery period, we conducted a single 10-minute recording after 6 hours without tracking the full temporal evolution. Combining both periods in one graph would not enhance the clarity or aesthetics, as the differences in the timing of recordings are too large. We prefer keeping them separate for a more accurate representation of the distinct phases of the experiment.

All raster plots of post hypoxia were in the original figures. However, for the figure showing the results of different temperatures in normoxia these raster plots were placed in a different location than the other figures. We adapted the figures making the layout more standardized.

4. It would be great to have some general viability data alongside the electrophysiological data to assess the general health of the neural cell populations in particular at the final time point.

We added additional experiments to evaluate the viability of the neuronal cultures after 24 and 48 hours of hypoxia for normothermic, hypothermic and hyperthermic neuronal networks, as well as 6 and 24 hours of recovery for normothermic and hypothermic neuronal networks, alongside our electrophysiological data. This will provide a comprehensive assessment of cell viability at the final time points.

Details are provided in the answer at point 1.

5. Line 442 in the conclusion “freezing the penumbra” is somewhat misleading as we are talking about a small change (37C to 34C) in temperature (not 0C), which would have significant adverse effects.

We adapted the conclusion leaving out “freezing the penumbra”, as we agree with the reviewer that it can be somewhat misleading.

Line 524-531: “*Our study demonstrates that hypothermia, applied during hypoxia, significantly enhances post-hypoxic functional recovery in neuronal networks, likely through the preservation of synaptic integrity and improved cell viability. The improvement in neuronal function, synaptic puncta and survival underscores hypothermia's potential as an effective neuroprotective strategy. These findings suggest that hypothermia could play a critical role in preserving brain function during the vulnerable period between arterial occlusion and recanalization in ischemic stroke patients, offering a promising therapeutic approach to mitigate irreversible neuronal damage.”*

6. The fact that hypothermia had such a strong effect on the control samples is somewhat concerning. Some loss in cell function (and most likely cell viability) is certainly expected (this is also shown in the Scimone et al as an in vitro example) but coming back to my point 1., without showing the long term, stable benefit of the application of hypothermia the overall study impact might be significantly diminished. Also, I was somewhat surprised to see, on the flip side, the almost positive effect of hyperthermia on the control cases - some more elaboration on what the authors think might be happening here within the context of theirs and other, similar in vitro culture systems will be useful.

We agree with the reviewer. We added data on cell viability including apoptosis after 24 and 48 hours of hypoxia for normothermic, hypothermic and hyperthermic neuronal networks, as well as 6 and 24 hours of recovery for normothermic and hypothermic neuronal networks (see answer to point 1). We also added results on synaptic puncta 6 and 24 hours post-hypoxia (see answer to point 2).

We also would like to thank the reviewer for his / her comment about the “almost positive effect of hyperthermia on the control cases”, which prompted us to re-evaluate our data. Upon review, we identified an outlier in the original dataset, which has now been corrected. We performed identification of outliers by employing Robust Regression and Outlier removal (ROUT) method with Q = 1%, meaning that data points that fell in the lower and upper 1% of the normal distribution were considered potential outliers. We have adjusted the figures accordingly, and the updated graphs make it clear that both hypothermia and hyperthermia are deleterious to normoxic neuronal networks.

References

1.	le Feber, J., et al., *Loss and recovery of functional connectivity in cultured cortical networks exposed to hypoxia.* J Neurophysiol, 2017. **118**(1): p. 394-403.

2.	Monteiro, S.P., et al., *Neuroprotective effect of hypoxic preconditioning and neuronal activation in a in vitro human model of the ischemic penumbra.* Journal of Neural Engineering, 2021. **18**(3): p. 036016.

3.	le Feber, J., et al., *Progression of Neuronal Damage in an In Vitro Model of the Ischemic Penumbra.* PLoS One, 2016. **11**(2): p. e0147231.

4.	Frega, M., et al., *Rapid Neuronal Differentiation of Induced Pluripotent Stem Cells for Measuring Network Activity on Micro-electrode Arrays.* JoVE (Journal of Visualized Experiments), 2017(119): p. e54900.

5.	Mossink, B., et al., *Cadherin-13 is a critical regulator of GABAergic modulation in human stem-cell-derived neuronal networks.* Mol Psychiatry, 2021.

6.	le Feber, J., et al., *Evolution of Excitation-Inhibition Ratio in Cortical Cultures Exposed to Hypoxia.* Front Cell Neurosci, 2018. **12**: p. 183.

7.	Feng, Z., et al., *Temperature-dependent structural plasticity of hippocampal synapses.* Front Cell Neurosci, 2022. **16**: p. 1009970.

8.	Wang, J., et al., *Differential fall in ATP accounts for effects of temperature on hypoxic damage in rat hippocampal slices.* J Neurophysiol, 2000. **83**(6): p. 3462-72.

9.	Takata, T., M. Nabetani, and Y. Okada, *Effects of hypothermia on the neuronal activity, [Ca2+]i accumulation and ATP levels during oxygen and/or glucose deprivation in hippocampal slices of guinea pigs.* Neurosci Lett, 1997. **227**(1): p. 41-4.

10.	Yager, J.Y. and J. Asselin, *Effect of mild hypothermia on cerebral energy metabolism during the evolution of hypoxic-ischemic brain damage in the immature rat.* Stroke, 1996. **27**(5): p. 919-25; discussion 926.

11.	Amorim, P., J.E. Cottrell, and I.S. Kass, *Effect of small changes in temperature on CA1 pyramidal cells from rat hippocampal slices during hypoxia: implications about the mechanism of hypothermic protection against neuronal damage.* Brain Res, 1999. **844**(1-2): p. 143-9.

12.	Yang, F. and J. Zheng, *High temperature sensitivity is intrinsic to voltage-gated potassium channels.* Elife, 2014. **3**: p. e03255.

13.	Kriegeskorte, S., et al., *Cold and warmth intensify pain-linked sodium channel gating effects and persistent currents.* J Gen Physiol, 2023. **155**(9).

14.	Graham, B.A., A.M. Brichta, and R.J. Callister, *Recording temperature affects the excitability of mouse superficial dorsal horn neurons, in vitro.* J Neurophysiol, 2008. **99**(5): p. 2048-59.
